# Influence of natal habitat preference on habitat selection during extra‐home range movements in a large ungulate

**DOI:** 10.1002/ece3.9794

**Published:** 2023-02-03

**Authors:** Nathan D. Hooven, Matthew T. Springer, Clayton K. Nielsen, Eric M. Schauber

**Affiliations:** ^1^ School of the Environment Washington State University Pullman Washington USA; ^2^ Department of Forestry and Natural Resources University of Kentucky Lexington Kentucky USA; ^3^ Cooperative Wildlife Research Laboratory and Department of Forestry Southern Illinois University Carbondale Carbondale Illinois USA; ^4^ Illinois Natural History Survey, Prairie Research Institute University of Illinois Urbana‐Champaign Champaign Illinois USA

**Keywords:** dispersal, excursion, habitat selection, landscape connectivity, movement, natal habitat preference induction

## Abstract

Natal habitat preference induction (NHPI) occurs when animals exhibit a preference for new habitat that is similar to that which they experienced in their natal environment, potentially leading to post‐dispersal success. While the study of NHPI is typically focused on post‐settlement home ranges, we investigated how this behavior may manifest during extra‐home range movements (EHRMs), both to identify exploratory prospecting behavior and assess how natal habitat cues may influence path selection before settlement. We analyzed GPS collar relocation data collected during 79 EHRMs made by 34 juvenile and subadult white‐tailed deer (*Odocoileus virginianus*) across an agricultural landscape with highly fragmented forests in Illinois, USA. We developed a workflow to measure multidimensional natal habitat dissimilarity for each EHRM relocation and fit step‐selection functions to evaluate whether natal habitat similarity explained habitat selection along movement paths. Across seasons, selection for natal habitat similarity was generally weak during excursive movements, but strong during dispersals, indicating that NHPI is manifested in dispersal habitat selection in this study system and bolstering the hypothesis that excursive movements differ functionally from dispersal. Our approach for extending the NHPI hypothesis to behavior during EHRMs can be applied to a variety of taxa and can expand our understanding of how individual behavioral variation and early life experience may shape connectivity and resistance across landscapes.

## Graphical abstract

Dispersing animals often rely upon cues from their early environment to maximize their post‐dispersal success. We found that white‐tailed deer select for familiar habitat along dispersal paths, suggesting important implications for animal movement and landscape connectivity.

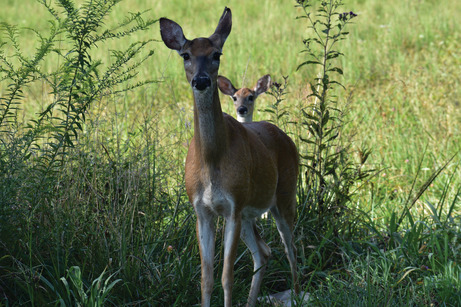

## 1 INTRODUCTION

Natal dispersal (hereafter, “dispersal”) can allow animals to limit inbreeding events (Clutton‐Brock, 1989; Wolff et al., 1988), avoid resource and breeding competition with conspecifics (Costello et al., 2008), and increase genetic connectivity and gene flow between subpopulations (Baguette et al., 2013). However, dispersal is a potentially risky event during which an animal can incur energetic and fitness costs while moving across hostile landscapes (Benoit et al., 2020; Bonte et al., 2012; Martinig et al., 2020). Thus, the configuration and connectivity of habitat along dispersal paths are critical in determining the duration and the cost of these movements (Baguette & Van Dyck, 2007). Dispersal is often sex‐biased in mammals (Dobson & Jones, 1985; Lawson Handley & Perrin, 2007) likely due to different fitness costs and benefits between the sexes (Costello et al., 2008; Greenwood, 1980; Packer & Pusey, 1987), and could lead to differing dispersal habitat use among demographic groups (Elliot et al., 2014). Additionally, these differences can be compounded by individual variation in dispersal behavior (Bowler & Benton, 2005; Clobert et al., 2009; Day et al., 2019; Doerr & Doerr, 2005).

One potential driver of variation in dispersal behavior is the preference exhibited by some animals to settle in habitat that is similar to what they experienced in their natal habitat after dispersal. Because dispersal can be costly, dispersing animals may attempt to increase their chances of post‐settlement success by choosing habitat that is similar to the habitat in their natal environment (Stamps & Swaisgood, 2007). This phenomenon is termed natal habitat preference induction (NHPI) and has been empirically tested in many animal taxa (Davis & Stamps, 2004). For example, dispersing Siberian flying squirrels (*Pteromys volans*) selected post‐dispersal habitat similar to their natal home range in terms of patch size and distance to forest edge (Selonen et al., 2007). Gray wolves (*Canis lupus*) making short‐distance dispersals tended to select new home ranges that were similar to their natal ranges (Sanz‐Pérez et al., 2018), and individual caribou (*Rangifer tarandus*) selected habitat attributes similarly between their natal and subadult life stages (Larue et al., 2018). Habitat preferences imprinted upon animals during early life can serve to modify their innate preferences (Burger & Gochfeld, 1990; Wecker, 1963), especially when faced with several choices later in life (Meredith, 1976). Thus, if behavioral flexibility afforded by learning and imprinting allows individuals to exploit resources they would innately avoid (Ditmer et al., 2015) or choose habitat similar to where they were previously successful (Mushinsky, 1976; Vogl et al., 2002), then previous experience could lead to improved individual survival and reproduction.

Because perceived habitat suitability drives the behavioral decisions of an animal during dispersal movements (Clobert et al., 2009), the ability to “sample” available habitat prior to dispersal may be important to post‐dispersal success (i.e., survival and reproduction; Badyaev et al., 1996). Animals often undertake exploratory movements outside of their natal home range prior to initiating dispersal, presumably to scout available habitat for breeding or establishing a new home range. This behavior, often called prospecting, is hypothesized to increase individual fitness after the establishment of a new breeding home range or territory as the animal will have gathered prior knowledge about available habitat and thus informing their decision on where to settle (Clobert et al., 2009; Oro et al., 2021). Prospecting movements are sensitive to habitat type and land cover (Cox & Kesler, 2012; Haughland & Larsen, 2004; Trainor et al., 2013), and thus can provide valuable information on the dispersal decision‐making process and can aid in predicting responses to environmental change (Delgado et al., 2014; Ponchon et al., 2013).

Selection of habitat along extra‐home range movement (EHRM) paths (including dispersals and exploratory movements) is important in understanding and predicting landscape connectivity for populations (Baguette et al., 2013; Benz et al., 2016; Ferrer & Harte, 1997; Killeen et al., 2014). Propensity to make prospecting EHRMs prior to dispersal may be tied to individual personality (Burkhalter et al., 2015; Debeffe et al., 2013), so it follows that habitat selection during both prospecting and dispersal EHRMs also may vary by individual (Wey et al., 2015). While studies of landscape connectivity attempt to link features and conditions of the environment with animal movement at a population level (Taylor et al., 1993; Tischendorf & Fahrig, 2000), individual behavioral variation may complicate inference of connectivity (Bélisle, 2005; Sullivan et al., 2021). Indeed, generalist species typically exhibit greater degrees of behavioral specialization (Bolnick et al., 2003; Carlson et al., 2021; Woo et al., 2008) and thus may adhere less to population‐level patterns of connectivity; additionally, this could be coupled with individual attraction to familiar landscape features (for an example with generalist species [wapiti, *Cervus canadensis*] see (Wolf et al., 2009)). We hypothesize that if settlement in a new habitat is mediated by natal habitat similarity, then habitat sampling behavior during prospecting and/or dispersal movements across the landscape may also show signs of selection for habitat characteristics that are familiar to those experienced in the natal habitat (Mabry & Stamps, 2008). By extending the NHPI hypothesis to include path selection along EHRMs, we may be better able to explain the use of habitat and corridors during these movements, where individuals attempt to minimize the perceived natal home range dissimilarity via habitat selection.

To test for evidence of NHPI during movements outside natal home ranges, we studied EHRM movement paths in a generalist ungulate species, the white‐tailed deer (*Odocoileus virginianus*), in a landscape consisting of fragmented forest patches in a matrix of row crop agriculture. Ungulates frequently make long‐distance movements between seasonal ranges or to settle in new habitats (Hoffman et al., 2006; Mueller et al., 2011). White‐tailed deer in particular often make long‐distance dispersal movements (Hygnstrom et al., 2008; Long et al., 2021; Moll et al., 2021), and several studies have shown that landscape features are important drivers of behavior during dispersals (Long et al., 2005; Lutz et al., 2016; Peterson et al., 2017). Deer also commonly make exploratory excursions or forays outside of their home ranges. These movements are often interpreted in the context of mate‐searching and conception (D'Angelo et al., 2004; Karns et al., 2011; Kolodzinski et al., 2010; Sullivan et al., 2017); however, excursions outside of the breeding season could be related to attempted dispersal (Lutz et al., 2016). Higher rates of excursive behavior have been correlated to future dispersal in roe deer (*Capreolus capreolus*) (Debeffe et al., 2013; Van Moorter et al., 2008), and as pre‐dispersal explorations are relatively common across mammalian taxa (Haughland & Larsen, 2004; Roper et al., 2003; Samelius et al., 2011; Selonen & Hanski, 2006), it is likely that some EHRMs serve to inform future dispersal in white‐tailed deer.

Here, we characterized dissimilarity to pre‐EHRM habitats and evaluated avoidance of dissimilar locations during excursive and dispersal movements, with the aim of quantifying the influence of NHPI on habitat selection during EHRMs. We used hourly relocation data from juvenile and subadult deer to identify EHRMs and measure the degree of habitat dissimilarity they experienced along movement paths. We tested for a link between movement path selection and familiarity of habitat composition and structure, to demonstrate whether deer make excursions to explore and scout potential new habitat while guiding their decisions based upon cues from their natal habitat. We hypothesized that both dispersers and individuals making exploratory, prospecting movements sample habitat based upon its similarity to what they experienced in their natal environment. We also hypothesized that habitat selection during EHRMs during the pre‐breeding/breeding season would be indifferent to natal habitat dissimilarity because these movements are likely to be aimed at efficiently finding and encountering potential mates.

## 2 MATERIALS AND METHODS

### 2.1 Study area

We captured deer for this study on land in and surrounding the Lake Shelbyville Project (LSP), a 13,892–ha area of public land administered and managed by the U.S. Army Corps of Engineers and the Illinois Department of Natural Resources in Moultrie and Shelby counties in central Illinois, USA. The LSP consisted of 4 separate managed areas, Lake Shelbyville (4451 ha), Eagle Creek State Park (921 ha), Wolf Creek State Park (832 ha), and Lake Shelbyville Fish and Wildlife Management Area (2604 ha). The LSP and surrounding area is dominated by row crop agriculture (45.0%; mostly corn and soybean), with a lower percentage (18.0%) of forested land concentrated around lakeshores and riparian areas, grassland (18.0%), and open water/wetlands (15.0%) (Anderson et al., 2015; Schauber et al., 2015; Springer, 2017). Long‐term mean temperatures ranged from 1.2°C in Jan–Mar, 17.5°C in Apr–Jun, 22.5°C in Jul–Sep, and 6.4°C Oct–Dec, with 15.9 mean total annual precipitation (Windsor, IL; MRCC, 2020).

### 2.2 Deer capture

During January–March 2011–2014, we captured white‐tailed deer using Clover traps (Clover, 1956), drop nets (Ramsey, 1968), rocket‐propelled nets (Hawkins et al., 1968), and free‐darting techniques. We chemically immobilized captured deer with a mixture of Telazol HCl (tiletamine HCl, 2 mg/kg, and zolazepam HCl, 4 mg/kg, Pfizer Animal Health) and Rompun (xylazine HCl, 2 mg/kg). We aged deer by examining tooth‐wear and development (Severinghaus, 1949), and fit an Iridium‐satellite uplink Global Positioning System (GPS) telemetry collar (Advanced Telemetry Systems, Inc) to all deer that were <22 months of age. We targeted juveniles (<12 months) and subadults (≥12 and <22 months) because these age classes are the most likely to disperse (Diefenbach et al., 2008; Nelson & Mech, 1984; Nixon et al., 1991), and we assumed these young deer were captured in/nearby their natal home range. After workup, we administered tolazoline (100 mg/kg) to aid in recovery from immobilization. All deer capture and handling methodology was approved by the Southern Illinois University Carbondale Institutional Animal Care and Use Committee (protocol #10–032), and followed guidelines set forth by the American Society of Mammalogists for the use of wild mammals in research (Sikes et al., 2011).

### 2.3 Defining EHRMs

To document EHRMs, we programmed GPS collars to send a relocation fix every hour for 8–10 months and then drop off via a release mechanism. We processed and analyzed data in either the R environment 3.6.2 (R Core Team, 2019) or ArcMap 10.6.1 (Esri). We used a conservative approach to censor inaccurate GPS relocations, removing all relocations with <5 horizontal dilution of precision (HDOP) if they were two‐dimensional fixes and all relocations with <6 HDOP if they were three‐dimensional fixes. To identify EHRMs, we employed a moving window approach as described by Jacobsen et al. (2020), defining a “pre‐EHRM home range” period as 30 days and the analysis window as the 2 days immediately following. Note that we calculated pre‐EHRM home ranges (1) to identify EHRMs and (2) to allow for comparison between movement path selection and familiar habitat (see Section 2.6). To characterize the pre‐EHRM home range, we fit Brownian bridge movement models (BBMM; Horne et al., 2007) with R packages “adehabitatLT” (version 0.3.24) and “adehabitatHR” (version 0.4.16; Calenge, 2019a, 2019b) and extracted the 95% isopleth as a polygon object. Then we determined which relocations in the analysis window fell outside this 95% pre‐EHRM home range and defined an EHRM as having (1) >1 relocation that was >500 m outside the pre‐EHRM home range and (2) >3 consecutive relocations outside the pre‐EHRM home range. We inspected visually to confirm each EHRM identified by the algorithm and identify its end point (Jacobsen et al., 2020). We separated dispersal movements from excursions when deer established a new, spatially disjoint area of restricted space use after making an EHRM; after all excursions deer returned to a location within their 95% BBMM isopleth.

### 2.4 Comparing used vs. available habitat during EHRMs

Habitat selection analyses based on animal relocations typically rely on comparing observed (used) relocations and other locations the animal could have used (available). We employed a step‐selection approach (Fortin et al., 2005; Thurfjell et al., 2014) to compare used and available habitat for each movement step (consecutive pair of relocations) during an EHRM. Once we identified an EHRM, we generated 50 random movement steps for each used step, all with the same starting point. We used R package “amt” version 0.1.3 (Signer et al., 2020) to generate each random step by drawing from a gamma distribution of step lengths and a von Mises distribution of turn angles based on EHRM movement data pooled across all individuals (Appendix S6). We chose to pool all individuals as some EHRMs were short (<5 relocations) and would not provide sufficient data to generate random steps.

### 2.5 Base habitat variables

We were interested in understanding how deer undertaking EHRMs select for overall features of deer habitat selected by the population at‐large vs. similarity to their specific natal habitat. We started by identifying four habitat features (forest cover, agriculture, streams, and primary/secondary roads) that prior research indicates are likely to be selected or avoided during movement paths (Gilbertson et al., 2022; Long et al., 2010; Nixon & Mankin, 2011; Springer, 2017), to use in base habitat models. We used ArcMap to reclassify the 30 m‐resolution National Land Cover Dataset (NLCD) 2011 raster to seven cover types (open water, wetland, forest, agriculture, open, developed, and other) for an extent defined by a 1‐km buffered bounding box around all deer relocations. Within this extent, we calculated the Euclidean distance from the forest and agriculture cover types for each pixel, resulting in two distance covariates (dForest and dAg). We used this approach because distance‐based habitat selection analyses are more robust to telemetry error than classification‐based approaches, provide information about selection for proximity to features that may not be explicitly used by the animal, and eliminate the need for inference based upon a reference land cover category (Conner et al., 2003; McNitt et al., 2020). We then retrieved stream polyline features from the 2019 USGS National Hydrography Dataset (https://www.usgs.gov/national‐hydrography/national‐hydrography‐dataset) and primary/secondary road polyline features from the U.S. Census Bureau TIGER/Line dataset (https://www.census.gov/geographies/mapping‐files/time‐series/geo/tiger‐line‐file.2011.html), clipped both layers to the study area extent, and generated Euclidean distance rasters at 30‐m resolutions. We used the extract() function in the “raster” package version 3.0–12 (Hijmans, 2020) to attribute covariate values from Euclidean distance rasters to each used and random location. We developed two hypotheses with these variables explaining base habitat selection during EHRMs (Table 1): selection for forested corridors along stream courses (Corridors) and avoidance of roads and row crops (Human Footprint).

**TABLE 1:** Hypotheses and covariates for base step selection function models.

Hypothesis	Ecological interpretation	Covariates	References
Corridors	Deer select for forested riparian corridors (along streams) when moving outside of their home range	dForest, dStream	Gilbertson et al. (2022), Springer (2017)
Human footprint	Deer avoid anthropogenic disturbance from roads as well as open agricultural fields when moving outside of their home range	dAg, dRoad	Gilbertson et al. (2022), Long et al. (2010), Peterson et al. (2017)
Global	Deer both select for forested riparian corridors and avoid anthropogenic features on the landscape when moving outside of their home range	dForest, dStream, dAg, dRoad	

### 2.6 Characterizing similarity/dissimilarity

We expanded upon the base habitat variables by incorporating metrics of similarity to habitat within deer home ranges. We assessed similarity on the basis of seven landscape variables that may influence deer space use and habitat selection: percentage forest, percentage developed, aggregation index, interspersion‐juxtaposition index, edge density, and patch richness density (Table 2; Anderson et al., 2011; Dechen Quinn et al., 2013; Walter et al., 2018). We chose these variables to broadly describe gradients in patch type composition, aggregation, and structure across the landscape while considering that deer likely perceive habitat cues at multiple spatial scales (Bowyer & Kie, 2006; Laforge et al., 2015; McGarigal et al., 2016). Accordingly, we calculated landscape variables with a moving window approach in FRAGSTATS version 4.2 (McGarigal & Marks, 1995) for four grain sizes with the following radii: 175 m, which was identified by Haus et al. (2020) as a minimum distance that a white‐tailed deer perceive the landscape; 250 m, corresponding to the mean step length during all EHRMs in our study (249.95 m); and twice the distances of each of these grain (350 m and 500 m) to encompass potential responses at wider spatial scales. This method generated 30‐m resolution rasters (*n* = 28) of each variable sampled at each grain size, and we extracted values from each for all used and random EHRM locations as with the base covariates.

**TABLE 2:** Landscape composition/configuration variables calculated at four spatial grains (175 m, 250 m, 350 m, 500 m), for which dissimilarity was calculated with the squared Mahalanobis distance.

Variable	Abbrev.	Explanation	References
% forest	for	Percentage of landscape in evergreen, mixed, and deciduous forest cover types	Anderson et al. (2011)
% developed	dev	Percentage of landscape in low‐, medium‐, and high‐intensity developed cover types	Anderson et al. (2011)
% open	open	Percentage of landscape in pasture, shrub‐scrub, and grassland cover types	Anderson et al. (2011)
Edge density	ED	Total length of the interface between unlike patch types divided by the area of the landscape	Anderson et al. (2011), Walter et al. (2018)
Interspersion‐juxtaposition index	IJI	Measurement of the intermixing of patch types	Dechen Quinn et al. (2013)
Patch richness density	PRD	Total number of distinct patch types divided by the area of the landscape	Dechen Quinn et al. (2013)
Aggregation index	AI	Number of like adjacencies divided by the total number of possible like adjacencies	Walter et al. (2018)

**FIGURE 1:**
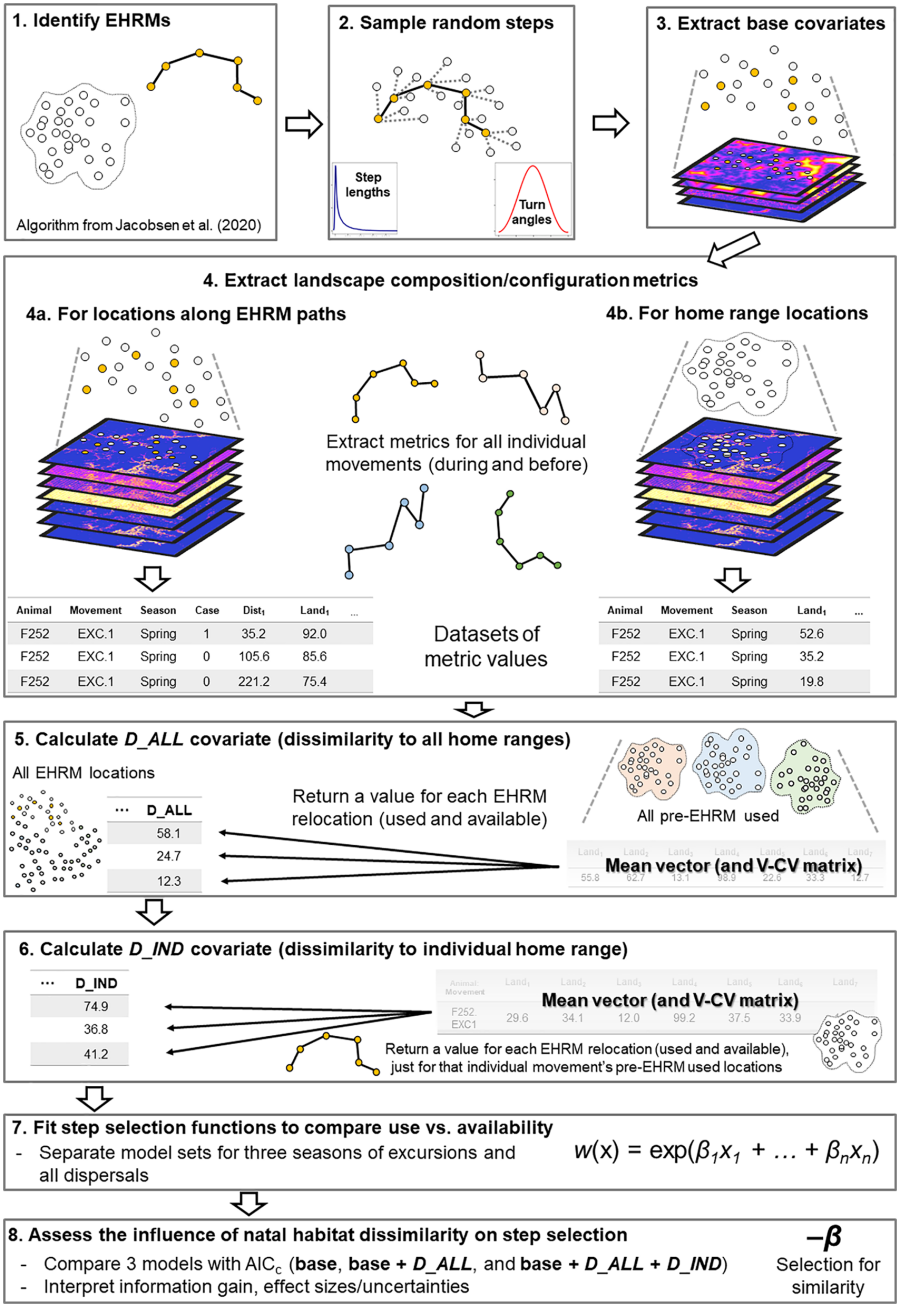
Diagram detailing the workflow we used to investigate the influence of natal habitat dissimilarity on habitat selection along extra‐home range movement paths.

We then used these landscape composition/configuration variables to calculate a multivariate index of dissimilarity between EHRM locations and home range locations. Multivariate methods have been applied to studies of NHPI between natal and settlement home ranges as a way to integrate several habitat variables simultaneously (Piper et al., 2013; Sanz‐Pérez et al., 2018), and as such we used the multidimensional Mahalanobis distance (Clark et al., 1993; Poor et al., 2020) to measure habitat dissimilarity. Each individual deer's natal home range contains features characterizing deer habitat in general (i.e., habitat features that are selected by the overall population), as well as the specific features unique to that home range. We addressed this problem by calculating two dissimilarity values for each EHRM location (observed or available): dissimilarity from that individual's pre‐EHRM home range (D_IND) and dissimilarity from home ranges of all deer in our study (D_ALL) (Figure 1).

Specifically, we calculated the squared Mahalanobis distance (*D* ^2^) of each EHRM location (used and available) from the distribution of locations in that movement's pre‐EHRM home range (excluding any previous EHRMs), using the seven variables calculated at each grain radius and producing one value for each EHRM location (resulting in covariates D_IND_175_, D_IND_250_, D_IND_350_, and D_IND_500_; Equation 1):

(1) $$D^{2}={\left(x–\mu \right)}^{T}C^{-1}\left(x–\mu \right)$$

where **x** is the observation (singular location) vector of landscape variables, **μ** is the mean vector for the movement's pre‐EHRM home range locations, and *C* ^−1^ is the inverse covariance matrix of the landscape variables. In our study, as *D* ^2^ values of EHRM locations increase, their dissimilarity to each home range increases. Because deer likely respond to landscape composition/configuration at the population level (i.e., a response to dissimilarity from individual‐specific pre‐EHRM home range locations may also capture an individual's response to features of overall deer habitat), we also calculated the *D* ^2^ between each EHRM location and the distribution of locations from all deer's pre‐EHRM home range locations for (D_ALL_175_, D_ALL_250_, D_ALL_350_, and D_ALL_500_).

While we consider these *D* ^*2*^ metrics to be appropriate for identifying disproportionate use of more familiar habitats during EHRMs, we acknowledge that they may be limited by the landscape metrics we chose. Although we included metrics that have been shown to influence deer space use, individual deer likely respond to a host of other variables that we either did not calculate or are not measured by the NLCD dataset. Studies of NHPI in other animals have not only evaluated habitat cover type and structure (Mabry & Stamps, 2008; Merrick & Koprowski, 2016) but also habitat quality/availability of specific resources (Selonen et al., 2007); because our spatial data serve only as proxies for resources, our analytical approach may overlook other important drivers of deer habitat selection.

### 2.7 Step‐selection analysis

**FIGURE 2:**
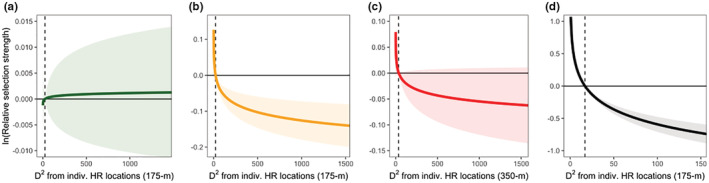
Model‐averaged predictions of the log‐relative selection strength (ln(RSS)), depicting selection (positive values) or avoidance (negative values) of squared Mahalanobis distance from movement‐specific pre‐EHRM relocations (D_IND) for three seasons of excursions (a: spring; b: summer; and c: fall) and dispersals (d). We calculated ln(RSS) with respect to “mean” locations, held all other covariates in the models at their mean value, and varied D_IND across its range for each season/movement type. Mean D_IND values are given by vertical dashed lines, and bands around predictions are 95% confidence limits.

We used step‐selection functions (SSFs; Fortin et al., 2005; Thurfjell et al., 2014) to model the relative intensity of space use, *w*(*x*), as a function of habitat covariates *x*_*1*_…*x*_*2*_ (Equation 2):

(2) $$w\left(x\right)=\exp\left(\beta_{1}x_{1}+\beta_{2}x_{2}+\ldots +\beta_{n}x_{n}\right)$$

where used locations (GPS relocations) are paired with endpoints of random steps (available locations) within strata via a conditional logistic regression framework. We used the method outlined by (Muff et al., 2020) to fit SSFs as generalized linear mixed effects models (GLMMs) using the glmmTMB function in the “glmmTMB” package version 0.2.3 (Magnusson et al., 2019), stratifying by step and including step length as a covariate (Avgar et al., 2016; Forester et al., 2009). We fit SSFs to 4 subsets of our EHRM dataset: three seasons of excursions depending upon when they occurred (spring: 1 Mar–31 May; summer: 1 Jun–31 Aug; fall: 1 Sep–30 Nov) and all dispersal movements pooled together. For each subset, we used a multi‐tiered model selection approach, first comparing functional forms (linear, quadratic, and natural log‐transformed) of each covariate with Akaike's information criterion corrected for small sample sizes (AIC_c_; Burnham & Anderson, 2002), to assess potentially nonlinear responses, and then retained the form that was most strongly supported (Lowrey et al., 2017; Ranglack et al., 2017). For the second tier, we compared three base model hypotheses (Table 1; Appendix S2–S5) using the best‐performing functional forms, advancing the top model to the next tier, in which we assessed the information gain afforded by the inclusion of the D_ALL and D_IND covariates (representing responses to overall deer habitat and natal habitat, respectively) along with their effect sizes and associated uncertainty. We evaluated the most informative functional form and spatial grain for both the D_ALL and D_IND covariates by comparing models including each term with AIC_c_ (Laforge et al., 2015; Lowrey et al., 2017). Note that in the case that the best performing forms of D_ALL and D_IND were highly correlated (*r* > 0.70), we selected the second‐ or third‐best fitting form to include in the final models. We included random slopes for each covariate to account for individual responses (Muff et al., 2020), unless random effect terms had very low (close to zero) variances leading to model convergence issues. We checked the variance inflation factors (VIFs) between main effects in each model to assess multicollinearity, considering VIFs <3 to be adequately low (Alston et al., 2020). In the case that multiple models were competitive (ΔAIC_c_ ≤ 2.00) and/or one model was not clearly the most informative, we performed model averaging (Burnham & Anderson, 2002; Cade, 2015) on the D_IND predictions when visualizing their effects (expressed as the log‐relative selection strength [RSS]; (Figure 2; Avgar et al., 2017)).

## 3 RESULTS

### 3.1 EHRMs

**FIGURE 3:**
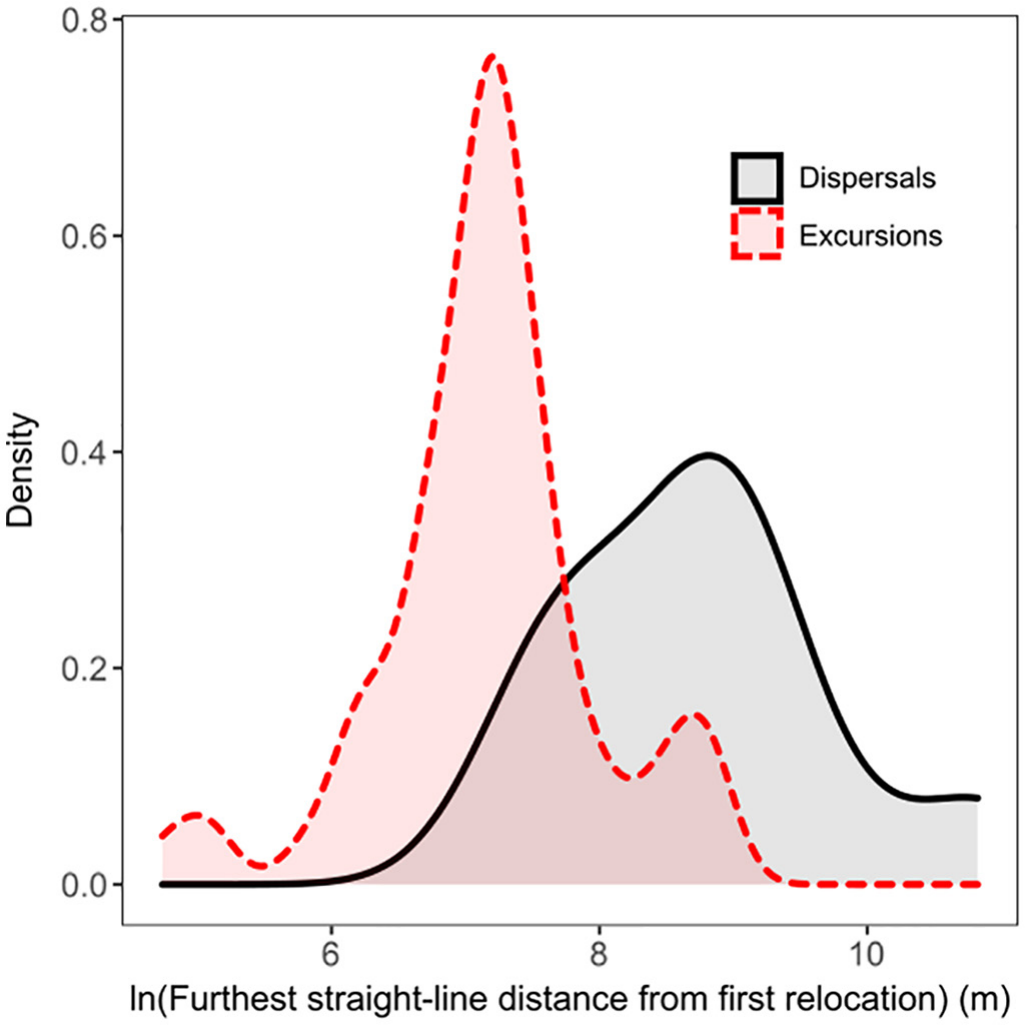
Density plots of the natural log‐transformed furthest straight‐line distance (m) from the first EHRM relocation for excursions (red dashed line) and dispersals (black solid line).

**FIGURE 4:**
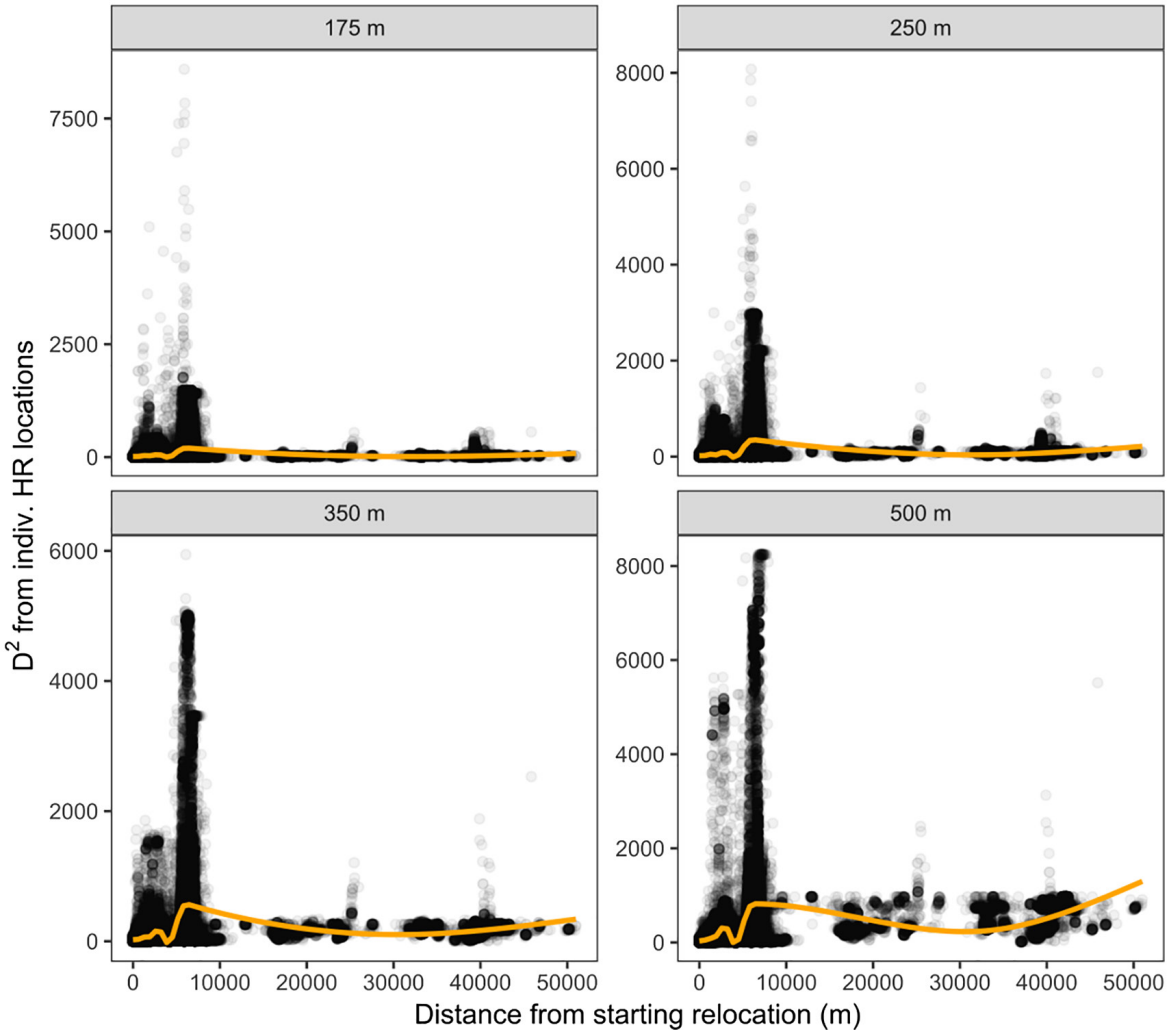
Relationship between the squared Mahalanobis distance (*D* ^*2*^) from individual pre‐movement home ranges (D_IND) and the straight‐line distance from each extra‐home range movement (EHRM) location and the first EHRM location. Gold lines are predictions from cubic regression splines depicting the nonlinear trend as distance increases. Each panel represents D_IND calculated at a different spatial grain.

We captured and fit GPS telemetry collars to 34 juvenile (22 male and nine female) and subadult (three female) deer that made EHRMs during our study. We identified and retained 79 EHRMs (nine dispersals and 70 excursions) for analysis, totaling 2472 movement steps. We documented the most excursions (*n* = 45) and dispersals (*n* = 8) in juvenile males (Appendix S1). The mean (±SE) number of excursions per deer was 2.3 ± 0.3 (range: 1–6). The mean (±SE) number of steps for excursions was 23.9 ± 6.1 (range: 3–341; median = 8) and the mean number of steps for dispersals was 88.9 ± 39.2 (range: 2–364; median = 54), while the mean time elapsed during excursions (in hours) was 27.4 ± 8.8 (range: 2.0–567.0; median = 9.0) and during dispersals was 88.6 ± 44.4 (range: 2.0–384.0; median = 56.0). Deer tended to move further from their home ranges during dispersals (Figure 3; range: 2026.2–50171.8 m; median = 6562.2 m) than excursions (range: 114.1–7099.0 m; median = 1327.1 m). However, the availability of dissimilar locations stayed mostly constant with increased distance from the pre‐EHRM home range (Figure 4). We classified excursions based upon which season they occurred: spring (*n* = 20), summer (*n* = 25), and fall (*n* = 25).

### 3.2 Step selection during EHRMs

Distance to forest cover and streams (representing the Corridors hypothesis) was an important correlate of step selection in every final model, although the top‐performing functional forms differed across seasons for dStream (Table 4). The Human Footprint hypothesis only received strong support for fall excursions (Table 4). For dispersal movements made year‐round, adding the ln(D_IND_175_) term dramatically improved model fit over the base‐only and the base + D_ALL models (Table 3), receiving all the model weight. The negative coefficient in this model (*β* [95% CI] = −0.27 [−0.42, −0.13]) suggested a strong avoidance response to natal habitat dissimilarity along dispersal paths (Figure 2). For spring excursions, the model which included the D_IND covariate received similar support (*ω*_*i*_ = 0.05) to the base‐only model (*ω*_*i*_ = 0.04) but much less support than the model that only included the base and D_ALL covariates (*ω*_*i*_ = 0.91), and the ln(D_IND_175_) selection coefficient was weakly positive (contrary to expectations; *β* = 0.01 [−0.3, 0.31]). For summer excursions, the base‐only model received the most support (*ω*_*i*_ = 0.65) although the selection coefficient for D_IND in the base + D_ALL + D_IND model (Δ AIC_c_ = 2.10, *ω*_*i*_ = 0.23) was negative (*β* = −0.23 [−0.43, −0.03]; Table 5). Deer appeared to be less sensitive to natal habitat dissimilarity during fall (1 Sep–30 Nov) excursions, again with the base‐only model providing the most information (*ω*_*i*_ = 0.52), and the ln(D_IND_350_) selection coefficient in the base + D_ALL + D_IND having wide uncertainty around its estimate (*β* = −0.07 [−0.29, 0.15]). Notably, outside of the spring excursion models, the effects of the D_ALL covariate tended to be weak (Table 5) and received little empirical support compared with the base model for summer and fall excursions.

**TABLE 3:** Final model selection tables for each excursion season and dispersal candidate set of generalized linear mixed model step selection functions. The number of parameters is given by *k*, while AIC_c_ is the Akaike's information criterion corrected for small sample sizes, ΔAIC_c_ is the difference from the top model, *ω*_*i*_ is the model weight, and LL is the log‐likelihood. “Base” refers to the initial set of landscape covariates, D_ALL refers to the squared Mahalanobis distance (*D* ^*2*^) from all deer home range relocations, and D_IND refers to the (*D* ^*2*^) from each individual's home range locations. Subscripts are the spatial grain of the covariates.

Season	Model structure	*k*	AICc	Δ AICc	*ω*_*i*_	LL
Dispersal	Base + D_ALL_175_ + ln(D_IND_175_)	7	18669.21	0.00	1.00	−9327.61
	Base + D_ALL_175_	8	18692.21	23.00	0.00	−9338.10
	Base	6	18696.77	27.56	0.00	−9342.39
Spring	Base + D_ALL_175_	11	16715.23	0.00	0.91	−8346.61
	Base + D_ALL_175_ + ln(D_IND_175_)	9	16721.02	5.80	0.05	−8351.51
	Base	7	16721.57	6.35	0.04	−8353.79
Summer	Base	6	11980.33	0.00	0.65	−5984.16
	Base + ln(D_ALL_250_) + ln(D_IND_175_)	10	11982.43	2.10	0.23	−5981.21
	Base + ln(D_ALL_250_)	8	11983.55	3.22	0.13	−5983.77
Fall	Base	8	10440.21	0.00	0.52	−5212.10
	Base + ln(D_ALL_175_) + ln(D_IND_350_)	11	10441.29	1.08	0.31	−5209.64
	Base + ln(D_ALL_175_)	10	10442.46	2.25	0.17	−5211.23

**TABLE 4:** Selection coefficients (*β* parameter estimates) and 95% confidence intervals from generalized linear mixed model step selection functions, given for base habitat covariates.

Season	Model	*ω*_*i*_	ln(dForest)	dStream	dStream^2^	ln(dStream)	ln(dAg)	ln(dRoad)	Step length
Dispersal	Base + All + Ind	1.00	−0.568 (−0.685, −0.451)			−0.252 (−0.335, −0.169)			0.215 (0.162, 0.269)
	Base + All	0.00	−0.577 (−0.721, −0.434)			−0.229 (−0.360, −0.098)			0.211 (0.157, 0.266)
	Base	0.00	−0.617 (−0.755, −0.480)			−0.220 (−0.353, −0.088)			0.209 (0.155, 0.264)
Spring	Base + All	0.91	−0.629 (−0.810, −0.449)	0.466 (0.106, 0.825)	−0.814 (−1.275, −0.353)				0.027 (−0.059, 0.113)
	Base + All + Ind	0.05	−0.605 (−0.767, −0.443)	0.481 (0.128, 0.834)	−0.796 (−1.251, −0.341)				0.031 (−0.055, 0.117)
	Base	0.04	−0.613 (−0.811, −0.416)	0.459 (0.101, 0.816)	−0.805 (−1.266, −0.345)				0.025 (−0.061, 0.111)
Summer	Base	0.65	−0.571 (−0.960, −0.181)	−0.202 (−0.538, 0.134)					0.049 (−0.050, 0.147)
	Base + All + Ind	0.23	−0.518 (−0.922, −0.115)	−0.223 (−0.566, 0.120)					0.053 (−0.045, 0.151)
	Base + All	0.13	−0.554 (−0.947, −0.161)	−0.202 (−0.537, 0.134)					0.048 (−0.050, 0.147)
Fall	Base	0.52	−0.519 (−0.781, −0.257)	0.113 (−0.175, 0.400)			0.186 (−0.014, 0.386)	0.222 (−0.082, 0.526)	0.200 (0.118, 0.283)
	Base + All + Ind	0.31	−0.460 (−0.73, −0.191)	0.097 (−0.192, 0.387)			0.218 (0.010, 0.426)	0.218 (−0.086, 0.522)	0.203 (0.120, 0.285)
	Base + All	0.17	−0.471 (−0.744, −0.198)	0.109 (−0.184, 0.401)			0.222 (0.016, 0.429)	0.217 (−0.087, 0.522)	0.199 (0.117, 0.282)

**TABLE 5:** Selection coefficients (*β* parameter estimates) and 95% confidence intervals from generalized linear mixed model step selection functions, given for squared Mahalanobis distance (*D* ^*2*^) from all individuals' pre‐extra‐home range movement (EHRM) relocations (D_ALL) and movement‐specific pre‐EHRM relocations (D_IND).

Season	Model	*ω*_*i*_	D_ALL_175_	ln(D_ALL_175_)	ln(D_ALL_250_)	ln(D_IND_175_)	ln(D_IND_350_)
Dispersal	Base + All + Ind	1.00	0.048 (−0.074, 0.170)			−0.274 (−0.416, −0.131)	
	Base + All	0.00	−0.151 (−0.286, −0.016)				
	Base	0.00					
Spring	Base + All	0.91	0.061 (−0.225, 0.346)				
	Base + All + Ind	0.05	−0.054 (−0.231, 0.123)			0.009 (−0.295, 0.312)	
	Base	0.04					
Summer	Base	0.65					
	Base + All + Ind	0.23			0.049 (−0.133, 0.231)	−0.230 (−0.430, −0.031)	
	Base + All	0.13			−0.068 (−0.217, 0.082)		
Fall	Base	0.52					
	Base + All + Ind	0.31		−0.075 (−0.231, 0.081)			−0.070 (−0.293, 0.153)
	Base + All	0.17		−0.097 (−0.204, 0.046)			

## 4 DISCUSSION

Dispersing animals often use cues in unfamiliar areas that recall aspects of their natal habitat (Benard & McCauley, 2008). Prospecting movements outside of an animal's natal home range may serve to inform subsequent dispersal (Burkhalter et al., 2015; Debeffe et al., 2013), but to our knowledge, a link between these movements and a preference for natal habitat similarity has not been demonstrated. We found strong selection for natal habitat similarity by white‐tailed deer during dispersal but not during other movements outside the natal home range, perhaps related to the enhanced fitness stakes of dispersal compared with other EHRMs. These findings are not consistent with the hypothesis that excursions primarily function to inform future dispersals (i.e., prospecting).

The strong effect of natal habitat dissimilarity on dispersal movement habitat selection compared to those for excursions demonstrates this difference between EHRMs of contrasting outcomes (settlement vs. return). Of course, this does not eliminate the likelihood that some excursions that we documented allowed individuals to gather information about the suitability of potential future habitat. Variability in the selection for similarity across individuals and movements could contribute to weak overall responses to the D_IND variable, especially during spring and fall excursions. While NHPI itself leads to individual variation in habitat selection, animals likely express heterogeneity in their preference for natal habitat cues, perhaps related to the quality of their natal habitats (Stamps et al., 2009). Given that the decision to leave a home range is influenced by many intrinsic and extrinsic factors, individuals may need to be flexible in how and where they move across the landscape.

Furthermore, deer seeking mates outside their home ranges may not select for landscape features indicative of habitat quality as strongly as during the rest of the year, which likely explains the weak overall effect of D_IND on space use during fall excursions. Mate searching behavior is common in both sexes of white‐tailed deer (Karns et al., 2011; Kolodzinski et al., 2010; Sullivan et al., 2017) and, while a majority of fall excursions were made by juveniles in our study (20/25, 80.0%), first year breeding is well documented in this species (Rhodes et al., 1986; Schultz & Johnson, 1992) and is likely related to high nutritional planes resulting from agricultural food subsidies (Haugen, 1975). Given the extensive corn‐soybean availability in our study area, it is probable that many juvenile deer in our study were sexually mature going into their first breeding season. With hunting seasons occurring during the autumn (October for archery and late November for firearm), disturbance resulting from hunting could trigger EHRMs as well (Sunde et al., 2009; Vercauteren & Hygnstrom, 1998), in which case excursions are evasive rather than exploratory in nature.

While animals may rely upon natal habitat cues for choosing a new home range, disconnects between perceived and actual habitat quality may lead to ecological traps and illustrate the potentially maladaptive nature of natal habitat preference (Fletcher et al., 2015; Larue et al., 2018; Piper et al., 2013). We were unable to assess the fitness consequences of individual preference in our study, but given that differences in habitat use can lead to varying survival and reproductive outcomes in cervids (Abernathy et al., 2022; Haus et al., 2020; Ofstad et al., 2020), natal‐biased dispersal in our study system could be correlated with success. If, as a strategy, NHPI is as or more likely to lead to negative fitness outcomes than positive ones (i.e., an ecological trap), then prospecting may not be worth its potential costs (Delgado et al., 2014). This could explain the weak responses to natal habitat dissimilarity we observed during most excursions and suggests that NHPI manifests more strongly during true dispersal paths rather than exploratory movements that could inform future dispersal.

Additionally, the spatial scale of movement and perception likely plays a role in the benefits gained from NHPI; dispersers use experience and memory from their natal habitat to estimate habitat quality (Stamps & Davis, 2006) so an individual's ability to perceive the landscape will inevitably affect the quality of its post‐settlement range. Sampling entire areas that will encompass a future home range is unlikely during relatively short dispersal movements, so smaller‐scale landscape cues must be needed. In our study, we tested several grain sizes to approximate the true perception distance, while acknowledging that landscape structure and other environmental conditions affect this distance (Olden et al., 2004). It is important to note that the most explanatory grains (e.g., the 175‐m radius leading to a 0.096 km^2^ area) covered only a small portion of a typical deer's home range in this region (mean home range size for subadult females during the pre‐breeding season: 1.10 km^2^; Nixon et al., 1991), and thus without more extensive sampling a deer likely would not be able to estimate the range's quality. Thus, dispersal can be a process that can take an extended period of time (Doerr & Doerr, 2005; Roper et al., 2003), and long search times and potential costs could cause individuals to settle for habitat of lower (perceived) quality the longer they search (Stamps et al., 2005). Habitat cues are perceived at a particular spatial scale (Webb et al., 2009), and while they may provide the disperser with the most information as possible during a brief EHRM, limitations imposed by perceptual ranges (Delgado et al., 2014; Pe'er & Kramer‐Schadt, 2008; Zollner & Lima, 1999) coupled with non‐ideal NHPI could reduce the animal's post‐dispersal success.

Our results also have important implications for modeling landscape connectivity, particularly with regard to the level of habitat suitability tolerated by dispersing animals. Keeley et al. (2017) suggest that animals readily move through low‐quality habitat when making EHRMs, and thus landscape resistance should not be assumed to be a linear inverse of suitability (Beier et al., 2008; Keeley et al., 2016; Trainor et al., 2013). In the case of our SSFs fit to dispersal movements, inclusion of the squared Mahalanobis distance (*D* ^*2*^) from all individuals' pre‐EHRM locations (D_ALL) only improved model fit slightly over the base model, while the individual movement‐specific *D* ^*2*^ covariate (D_IND) provided much more information. Deer thus appeared to be responding only weakly to a metric of population‐wide selected deer habitat (as measured during typical ranging behavior) during dispersals but responding strongly to familiar habitat cues. This outcome highlights the potential limitations of estimating landscape resistance using functions of suitability derived either from home range resource selection functions (RSFs) using all sampled individuals or RSFs fit to movement path relocations (Zeller et al., 2012), as neither approach traditionally examines individual variation due to NHPI. Indeed, individual habitat preferences may reduce the perceived habitat suitability across the landscape, thereby narrowing potential dispersal corridors for some individuals.

One possible limitation of our study is related to the difference in movement distance between excursions (relatively short) and dispersals (typically longer, including one individual that moved >50 km from its original home range). We acknowledge that some of the patterns we documented here, notably the lack of selection for natal habitat similarity during excursions, could be due to nearby habitat being on the whole more similar to where each deer originated from. Thus, selection for similarity might be less likely to manifest during these temporary EHRMs because excursing individuals experience less unfamiliar habitat than dispersers. However, availability of dissimilar habitats did not tend to increase as distance from the pre‐EHRM home range increased, especially for finer scales, so we consider our assumption that deer making both excursions and dispersals encountered similar amounts of unfamiliar areas appropriate. Our inference is, of course, limited by the relatively small number of dispersal events (*n* = 9) we documented, and further study should be conducted on larger datasets to corroborate the results we present here.

Dispersal is a challenging biotic process to study (Kokko & López‐Sepulcre, 2006; Nathan, 2001), and understanding the landscape‐level drivers of dispersal movements is critical in large‐scale habitat conservation and management planning. By using a metric of natal habitat dissimilarity to investigate the selection for familiar habitat cues during EHRMs, our approach bridges the gap between the study of NHPI and landscape connectivity. The methods we present here can accommodate tracking data from a variety of taxa and movement behaviors and have the potential to deepen our understanding of dispersal, landscape resistance, and gene flow in animal populations. Our framework can assist in elucidating the extent and drivers of individual variation in dispersal habitat selection, a key component in understanding how animals make decisions on where to go and where to settle (Baguette et al., 2013; Merrick & Koprowski, 2017).

## AUTHOR CONTRIBUTIONS

**Nathan D. Hooven:** Conceptualization (equal); data curation (lead); formal analysis (lead); methodology (lead); visualization (lead); writing – original draft (lead). **Matthew T. Springer:** Conceptualization (equal); investigation (lead); methodology (supporting); writing – review and editing (equal). **Clay K. Nielsen:** Conceptualization (equal); funding acquisition (equal); project administration (equal); resources (equal); writing – review and editing (equal). **Eric M. Schauber:** Funding acquisition (equal); methodology (supporting); project administration (equal); resources (equal); writing – review and editing (equal).

## CONFLICT OF INTEREST STATEMENT

The authors declare no competing interests.

## Supporting information


Appendix S1

## Data Availability

Data generated from and used in analysis for this study are available via Zenodo: http://doi.org/10.5281/zenodo.7315810. R scripts are available from: https://github.com/nhooven/deer‐natal‐habitat.
