# Supplementary material for: Influence of natal habitat preference on habitat selection during extra‐home range movements in a large ungulate
Source: Ecol Evol. 2023 Feb 3;13(2):e9794. doi: 10.1002/ece3.9794 (PMC9897958; doi:10.1002/ece3.9794)

**Appendix 1**

Frequency of extra-home range movements EHRMs by type, sex, age, and season.

| Type | Sex | Age | Season | Frequency |
| --- | --- | --- | --- | --- |
| Excursion | Male | Juvenile | Spring | 15 |
|  |  |  | Summer | 19 |
|  |  |  | Fall | 11 |
|  | Female | Juvenile | Spring | 3 |
|  |  |  | Summer | 5 |
|  |  |  | Fall | 9 |
|  |  | Subadult | Spring | 2 |
|  |  |  | Summer | 1 |
|  |  |  | Fall | 5 |
| Dispersal | Male | Juvenile | Spring | 2 |
|  |  |  | Summer | 1 |
|  |  |  | Fall | 5 |
|  | Female | Juvenile | Spring | 1 |

**Appendix 2**

Spring model selection results for covariate optimization, including functional forms for base covariates (dAg, dForest, dRoad, dStream), three base hypotheses (Corridors, Human, and Global), and functional forms/spatial grains for D_ALL and D_IND covariates.

| Variable | Model | Δ AICc | *ω_i_* |
| --- | --- | --- | --- |
| dAg | Quadratic | 0.00 | 0.93 |
|  | Pseudothreshold | 5.10 | 0.07 |
|  | Linear | 43.66 | 0.00 |
| dForest | Pseudothreshold | 0.00 | 1.00 |
|  | Quadratic | 32.69 | 0.00 |
|  | Linear | 42.26 | 0.00 |
| dRoad | Linear | 0.00 | 0.44 |
|  | Pseudothreshold | 0.28 | 0.38 |
|  | Quadratic | 1.84 | 0.18 |
| dStream | Quadratic | 0.00 | 0.91 |
|  | Linear | 4.53 | 0.09 |
|  | Pseudothreshold | 32.69 | 0.00 |
| Base | Corridors | 0.00 | 0.61 |
|  | Global | 0.91 | 0.39 |
|  | Human | 138.76 | 0.00 |
| D_ALL | 175 pseudothreshold | 0.00 | 1.00 |
|  | 175 quadratic | 29.21 | 0.00 |
|  | 175 | 40.97 | 0.00 |
|  | 250 pseudothreshold | 41.84 | 0.00 |
|  | 350 pseudothreshold | 49.45 | 0.00 |
|  | 250 quadratic | 49.57 | 0.00 |
|  | 250 | 49.61 | 0.00 |
|  | 500 pseudothreshold | 50.31 | 0.00 |
|  | 350 | 50.78 | 0.00 |
|  | 500 | 51.10 | 0.00 |
|  | 350 quadratic | 52.19 | 0.00 |
|  | 500 quadratic | 52.83 | 0.00 |
| D_IND | 175 pseudothreshold | 0.00 | 0.98 |
|  | 500 quadratic | 7.89 | 0.02 |
|  | 500 | 14.19 | 0.00 |
|  | 350 quadratic | 20.43 | 0.00 |
|  | 350 | 20.96 | 0.00 |
|  | 250 pseudothreshold | 22.15 | 0.00 |
|  | 350 pseudothreshold | 24.40 | 0.00 |
|  | 500 pseudothreshold | 25.66 | 0.00 |
|  | 175 | 25.96 | 0.00 |
|  | 175 quadratic | 27.37 | 0.00 |
|  | 250 | 27.96 | 0.00 |
|  | 250 quadratic | 29.77 | 0.00 |

**Appendix 3**

Summer model selection results for covariate optimization, including functional forms for base covariates (dAg, dForest, dRoad, dStream), three base hypotheses (Corridors, Human, and Global), and functional forms/spatial grains for D_ALL and D_IND covariates.

| Variable | Model | Δ AICc | *ω_i_* |
| --- | --- | --- | --- |
| dAg | Pseudothreshold | 0.00 | 0.90 |
|  | Quadratic | 4.57 | 0.09 |
|  | Linear | 8.48 | 0.01 |
| dForest | Pseudothreshold | 0.00 | 1.00 |
|  | Quadratic | 21.51 | 0.00 |
|  | Linear | 30.72 | 0.00 |
| dRoad | Pseudothreshold | 0.00 | 0.50 |
|  | Linear | 0.89 | 0.32 |
|  | Quadratic | 2.08 | 0.18 |
| dStream | Linear | 0.00 | 0.68 |
|  | Quadratic | 1.48 | 0.32 |
|  | Pseudothreshold | 15.04 | 0.00 |
| Base | Corridors | 0.00 | 0.77 |
|  | Global | 2.41 | 0.23 |
|  | Human | 70.34 | 0.00 |
| D_ALL | 250 pseudothreshold | 0.00 | 0.51 |
|  | 175 pseudothreshold | 0.74 | 0.35 |
|  | 250 quadratic | 4.83 | 0.05 |
|  | 350 pseudothreshold | 5.55 | 0.03 |
|  | 175 quadratic | 5.71 | 0.03 |
|  | 500 | 7.99 | 0.01 |
|  | 175 | 8.00 | 0.01 |
|  | 250 | 8.60 | 0.01 |
|  | 500 quadratic | 9.96 | 0.00 |
|  | 500 pseudothreshold | 10.31 | 0.00 |
|  | 350 | 10.35 | 0.00 |
|  | 350 quadratic | 11.15 | 0.00 |
| D_IND | 175 pseudothreshold | 0.00 | 0.98 |
|  | 350 pseudothreshold | 8.14 | 0.02 |
|  | 250 pseudothreshold | 16.31 | 0.00 |
|  | 500 | 18.44 | 0.00 |
|  | 500 pseudothreshold | 19.98 | 0.00 |
|  | 175 | 20.10 | 0.00 |
|  | 250 | 20.23 | 0.00 |
|  | 500 quadratic | 20.34 | 0.00 |
|  | 350 | 20.55 | 0.00 |
|  | 350 quadratic | 20.93 | 0.00 |
|  | 175 quadratic | 21.70 | 0.00 |
|  | 250 quadratic | 21.83 | 0.00 |

**Appendix 4**

Fall model selection results for covariate optimization, including functional forms for base covariates (dAg, dForest, dRoad, dStream), three base hypotheses (Corridors, Human, and Global), and functional forms/spatial grains for D_ALL and D_IND covariates.

| Variable | Model | Δ AICc | | *ω_i_* |
| --- | --- | --- | --- | --- |
| dAg | Pseudothreshold | 0.00 | 1.00 | |
|  | Quadratic | 13.80 | 0.00 | |
|  | Linear | 26.21 | 0.00 | |
| dForest | Pseudothreshold | 0.00 | 0.89 | |
|  | Quadratic | 4.26 | 0.11 | |
|  | Linear | 25.95 | 0.00 | |
| dRoad | Pseudothreshold | 0.00 | 0.62 | |
|  | Quadratic | 1.92 | 0.24 | |
|  | Linear | 2.85 | 0.15 | |
| dStream | Linear | 0.00 | 0.51 | |
|  | Pseudothreshold | 1.15 | 0.29 | |
|  | Quadratic | 1.93 | 0.20 | |
| Base | Global | 0.00 | 0.89 | |
|  | Corridors | 4.11 | 0.11 | |
|  | Human | 27.24 | 0.00 | |
| D_ALL | 175 pseudothreshold | 0.00 | 0.77 | |
|  | 175 quadratic | 4.47 | 0.08 | |
|  | 175 | 7.03 | 0.02 | |
|  | 500 quadratic | 7.04 | 0.02 | |
|  | 250 pseudothreshold | 7.52 | 0.02 | |
|  | 350 pseudothreshold | 7.84 | 0.02 | |
|  | 500 pseudothreshold | 7.90 | 0.01 | |
|  | 250 | 7.99 | 0.01 | |
|  | 500 | 8.15 | 0.01 | |
|  | 350 | 8.18 | 0.01 | |
|  | 350 quadratic | 8.45 | 0.01 | |
|  | 250 quadratic | 9.73 | 0.01 | |
| D_IND | 350 pseudothreshold | 0.00 | 0.30 | |
|  | 500 pseudothreshold | 0.48 | 0.24 | |
|  | 175 pseudothreshold | 1.69 | 0.13 | |
|  | 500 quadratic | 2.27 | 0.10 | |
|  | 175 | 3.43 | 0.05 | |
|  | 350 | 4.24 | 0.04 | |
|  | 250 | 4.36 | 0.03 | |
|  | 250 pseudothreshold | 4.38 | 0.03 | |
|  | 500 | 4.67 | 0.03 | |
|  | 175 quadratic | 5.08 | 0.02 | |
|  | 350 quadratic | 5.94 | 0.02 | |
|  | 250 quadratic | 6.22 | 0.01 | |

**Appendix 5**

Dispersal model selection results for covariate optimization, including functional forms for base covariates (dAg, dForest, dRoad, dStream), three base hypotheses (Corridors, Human, and Global), and functional forms/spatial grains for D_ALL and D_IND covariates.

| Variable | Model | Δ AICc | *ω_i_* |
| --- | --- | --- | --- |
| dAg | Pseudothreshold | 0.00 | 1.00 |
|  | Quadratic | 26.78 | 0.00 |
|  | Linear | 41.44 | 0.00 |
| dForest | Pseudothreshold | 0.00 | 1.00 |
|  | Quadratic | 27.51 | 0.00 |
|  | Linear | 62.93 | 0.00 |
| dRoad | Pseudothreshold | 0.00 | 0.60 |
|  | Linear | 1.43 | 0.29 |
|  | Quadratic | 3.33 | 0.11 |
| dStream | Pseudothreshold | 0.00 | 1.00 |
|  | Quadratic | 20.73 | 0.00 |
|  | Linear | 43.95 | 0.00 |
| Base | Corridors | 0.00 | 0.77 |
|  | Global | 2.42 | 0.23 |
|  | Human | 96.75 | 0.00 |
| D_ALL | 175 pseudothreshold | 0.00 | 0.75 |
|  | 250 | 3.69 | 0.12 |
|  | 250 quadratic | 5.07 | 0.06 |
|  | 175 | 6.44 | 0.03 |
|  | 175 quadratic | 6.90 | 0.02 |
|  | 250 pseudothreshold | 7.67 | 0.02 |
|  | 350 | 22.66 | 0.00 |
|  | 350 pseudothreshold | 23.84 | 0.00 |
|  | 350 quadratic | 24.51 | 0.00 |
|  | 500 pseudothreshold | 24.70 | 0.00 |
|  | 500 | 28.48 | 0.00 |
|  | 500 quadratic | 30.38 | 0.00 |
| D_IND | 175 pseudothreshold | 0.00 | 0.97 |
|  | 175 | 8.22 | 0.02 |
|  | 175 quadratic | 8.44 | 0.01 |
|  | 250 pseudothreshold | 18.31 | 0.00 |
|  | 250 | 21.73 | 0.00 |
|  | 250 quadratic | 22.47 | 0.00 |
|  | 350 pseudothreshold | 29.36 | 0.00 |
|  | 350 | 31.50 | 0.00 |
|  | 350 quadratic | 33.49 | 0.00 |
|  | 500 pseudothreshold | 41.66 | 0.00 |
|  | 500 | 42.23 | 0.00 |
|  | 500 quadratic | 44.20 | 0.00 |

**Appendix 6**

Distributions of step lengths (gamma distribution) and turn angles (von Mises distribution) from extra-home range movements, used to draw random steps for step selection functions.


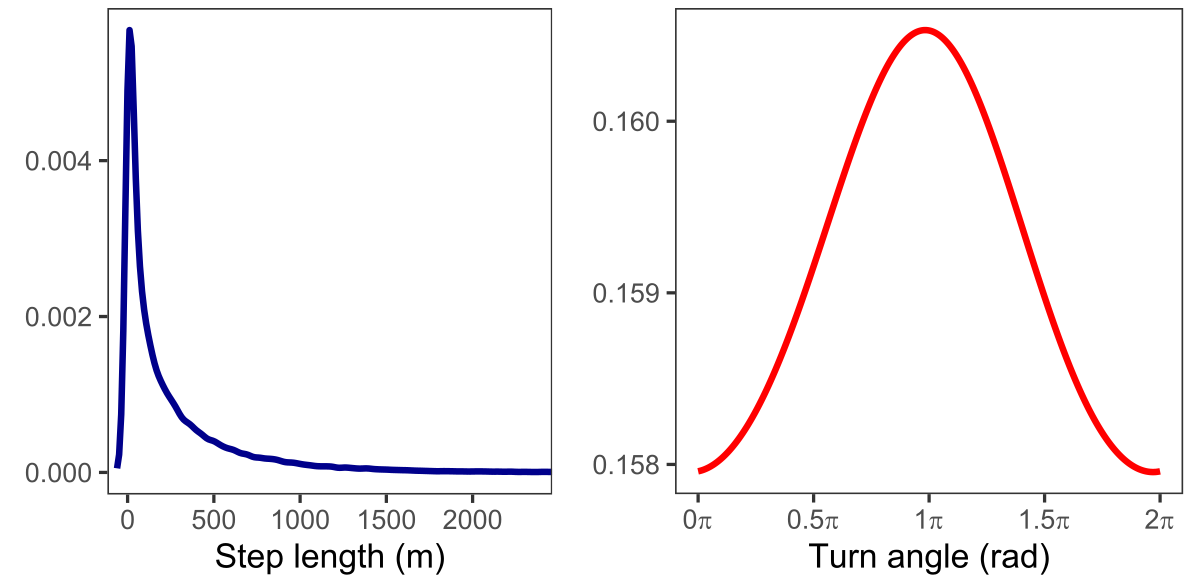


**Appendix 7**

Convex hull plots of individual specific pre-extra-home range movement (EHRM) home range relocations based upon seven metrics of landscape composition/configuration. Hulls are visualized in reduced dimensional space via results from principal components analyses conducted per buffer. These plots illustrate how habitat use during different pre-EHRM home ranges (each hull) differs from the overall pattern of habitat use.
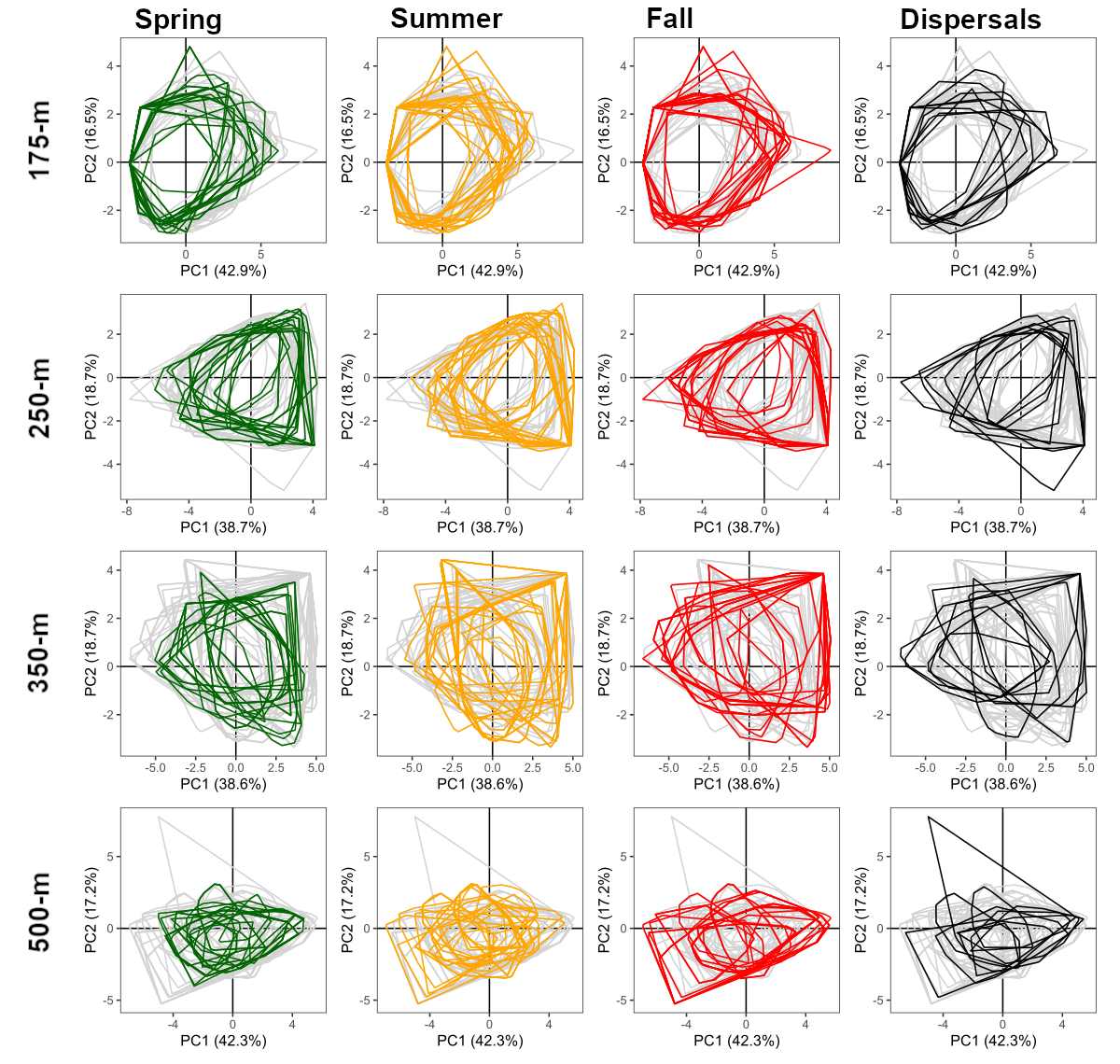

Supplement: Supplementary file 1 — Appendix S1 [file ECE3-13-e9794-s001.docx]
